# Effect of the intra-alveolar administration of dexamethasone on swelling, trismus, and pain after impacted lower third molar extraction: a randomized, double-blind clinical trial

**DOI:** 10.4317/medoral.24894

**Published:** 2021-09-25

**Authors:** Rogério Vera Cruz Ferro Marques, Luciana Salles Branco-de-Almeida, Daniele Meira Conde Marques, Izabel Cristina Vieira de Oliveira, Saulo José Figueiredo Mendes, Vandilson Pinheiro Rodrigues, Fernanda Ferreira Lopes

**Affiliations:** 1 Doctor, Post Graduate Program in Dentistry, School of Dentistry, Federal University of Maranhão; 2 Assistant Professor, Post Graduate Program in Dentistry, School of Dentistry, Federal University of Maranhão; 3 Assistant Professor, Department of Dentistry I, Federal University of Maranhão; 4 PhD student, Post Graduate Program in Dentistry, School of Dentistry, Federal University of Maranhão; 5 Assistant Professor, Department of Pharmacy, Ceuma University; 6 Associate Professor, Post Graduate Program in Dentistry, School of Dentistry, Federal University of Maranhão

## Abstract

**Background:**

To evaluate the efficacy of intra-alveolar administration of dexamethasone 4 mg in the control of edema, trismus, and pain resulting from the extraction of impacted lower third molars and the drug permeability through the oral mucosa by *in silico* prediction.

**Material and Methods:**

The randomized, double-blind, split-mouth clinical trial included patients who had both impacted lower third molars in equivalent positions. Hemiarches were divided into control side when dexamethasone was administered orally and experimental side when dexamethasone was administered using the intra-alveolar route. Patients were evaluated considering edema, trismus, and pain. The permeability of dexamethasone through the oral mucosa was assessed by *in silico* prediction. Student’s t-test was selected for comparative analysis of edema and trismus, and the chi-square test analyzed the distribution of postoperative pain between the sides.

**Results:**

There were no significant differences between the routes of administration in measuring symptoms between the pre and postoperative times (*p*>0.05). *In silico* prediction suggested that dexamethasone molecular characteristics facilitate intra-alveolar administration.

**Conclusions:**

Intra-alveolar administration had similar efficacy to oral administration in controlling symptoms of post-surgical inflammation of impacted lower third molars.

** Key words:**Molar, third, tooth, impacted, oral surgical procedures, dexamethasone.

## Introduction

The extraction of impacted lower third molars is a procedure that involves extensive tissue trauma and a considerable postoperative inflammatory response. Postoperative complications resulting from surgery include edema, trismus, and pain, which cause morbidity and affect patients’ quality of life (1). The surgical technique employed, the severity of impaction, the surgical procedure complications, and the inadequate pharmacological control (2) influence the intensity of symptoms.

Steroidal anti-inflammatory drugs have been widely used to control inflammation and postoperative signs/symptoms associated with the extraction of third molars and other oral surgeries (3,4), mainly blocking or inhibiting the action of inflammatory mediators (5). In this context, dexamethasone is a steroidal, adrenocortical (glucocorticoid) anti-inflammatory drug (AID) with high anti-inflammatory power, prolonged action, and low profile of side effects when used in the short term, with proven efficacy in controlling inflammation after extracting lower molar third parties (1), and is the second AID and first steroidal AID prescribed by dentists (6). Its anti-inflammatory actions vary and are mainly associated with the induction of the expression of the anti-inflammatory protein annexin-1 (7); reduced levels of prostaglandins, serotonin, bradykinins, cortisol, and lymphokines; and the suppressive effect on lymphocytes, monocytes, and eosinophils (8).

The oral route has been the most widely used, investigated, and convenient route of administration for dexamethasone, in the drug dosages of 4 or 8 mg (5). Administration one hour before the surgical procedure aims to obtain preemptive analgesia, reducing analgesics in the postoperative period (4). Previous studies have shown a beneficial effect in reducing inflammatory symptoms during a short postoperative period (9). In contrast, no protocol clearly defines the best route of administration for the control of inflammation after third molar surgery, as all methods have advantages and disadvantages (10).

Parenteral routes of administration of dexamethasone have also been considered (2,7,11) due to the enzymatic degradation in the gastrointestinal tract via the oral route (first-pass effect on the liver) and the need for patient cooperation. In this context, in a previous study, a powdered dexamethasone formulation applied inside the alveolus after surgery had similar efficacy in controlling inflammation signs/symptoms after extraction of included third molars when compared to submucosal injection of the drug (12). However, studies evaluating the effects of intra-alveolar administration of dexamethasone on signs/symptoms of post-tooth extraction inflammation of third molars remain scarce.

The topical intra-alveolar use of dexamethasone administered by the professional right after extraction (12) would also be convenient for the patient and would be yet another therapeutic option. The copious blood flow and mucosa permeability would facilitate drug absorption when applied to the oral mucosa/surgical wound (13). On the other hand, not all drugs have the physical-chemical characteristics necessary for administration through the oral mucosa (13,14), which requires evaluating dexamethasone in this regard.

This study evaluated the efficacy of topical intra-alveolar administration of dexamethasone used in a powder formulation (compared to the use of the same formulation orally), in the control of edema, trismus, and pain resulting from extraction by impacted third lower molars. Also, dexamethasone permeability through the oral mucosa was assessed using *in silico* prediction, a computational model predicting drug permeability in the oral mucosa based on molecular descriptors.

## Material and Methods

**Figure 1:**
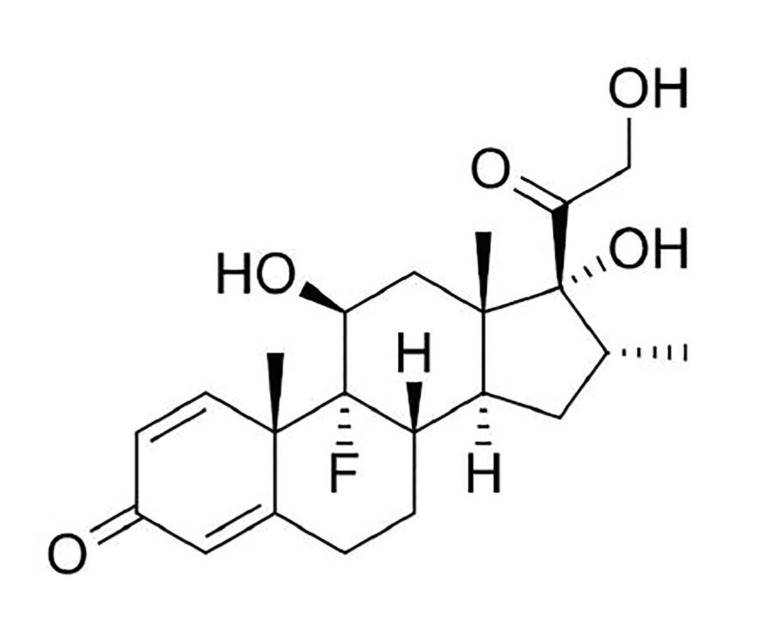
Chemical structure of dexamethasone.

A randomized, double-blind, split-mouth clinical trial was conducted. The study hypothesizes that the topical intra-alveolar administration of dexamethasone (Fig. 1) is as effective as the systemic oral route in the control of edema, trismus, and pain resulting from the extraction of impacted lower third molars. This study was submitted to and approved by the Research Ethics Committee (CAAE: 45361315.1.0000.5087).

Patients were recruited from a private clinic. The study included male and female patients aged 18-40 years with two impacted lower third molars, in equivalent positions according to the classification of Winter [1926] and Pell & Gregory [1933]. We excluded patients with any local or systemic changes that could influence the results, such as caries or advanced periodontal disease; pain in the third molar region; cysts or tumors; use of analgesics or anti-inflammatory drugs in the 15 days before the surgical procedure; hypersensitivity to the drugs used in the study; pregnancy or lactation and uncontrolled systemic health conditions such as systemic arterial hypertension, diabetes mellitus, and coagulation disorders; whose surgeries lasted more than an hour and those in which the time of surgery varied by more than five minutes between each side (15).

**Figure 2:**
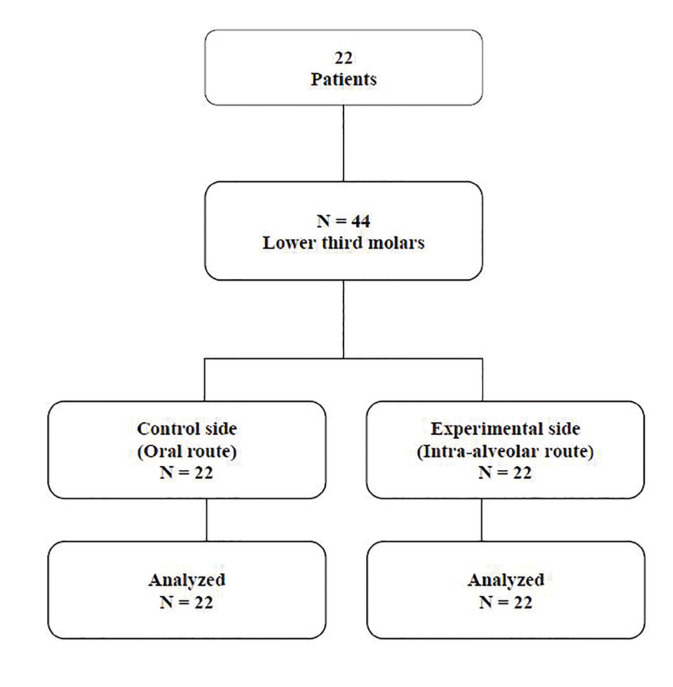
CONSORT flowchart.

The patients underwent two surgeries to extract the lower third molars, with a minimum interval of 30 days between each one. The hemiarches of each patient were divided into i) control side, when 4 mg dexamethasone (Fig. 2; Fagron, Brazil) was administered orally one hour before surgery, using a powder formulation contained in capsules; and ii) experimental side, when 4 mg dexamethasone was administered intraoperatively via the intra-alveolar route, using the same powder formulation.

The side to be operated on and the administration route were selected randomly, by lot, using a specific program (Excel; Microsoft, 2015) and under double-blind conditions. When the surgical procedure was performed on the experimental side, patients received, one hour before surgery, a placebo similar to dexamethasone administered orally. The evaluator and the patients were unaware of the administration route adopted on each side before evaluating the clinical parameters.

Before the surgical procedure, intra-oral antisepsis was performed by mouthwash, for one minute, with a 0.12% aqueous solution of chlorhexidine digluconate. A 2% aqueous solution of chlorhexidine digluconate was used for extra-oral antisepsis.

A 2% lidocaine solution with epinephrine 1: 100,000 (DFL, Rio de Janeiro, RJ, Brazil) was used for local anesthesia, due to regional block and subperiostatic infiltration, depending on the operated region (mandible), observing the maximum anesthetic dose, based on the volunteers’ body weight (16).

The access was performed through mucoperiosteal flaps, one distal and one mesial, to expose the bone that covers the impacted third molar. The detachment was done with a Molt detacher nº 9 (Quinelato, Rio Claro, SP, Brazil). Drills nº 5 and nº 703 were used, respectively, for osteotomy and odontosection, irrigated with 0.9% saline solution. After extraction, the site was inspected, regularized, and sutured with 5-0 nylon thread (Ethicon, Inc.). A 4 mg dexamethasone capsule was administered orally one hour before surgery on the control side; and on the experimental side, 4 mg dexamethasone powder was applied inside the alveolus before suturing.

The surgeries were performed by a single dentist specialized in maxillofacial surgery (R.V.C.F.M.). Patients received postoperative recommendations on cleaning the operated region with a mouthwash with 15 ml of 0.12% chlorhexidine digluconate aqueous solution every 12 hours until the return visit for suture removal seven days later. The volunteers were also instructed on local hemostatic measures, a semi-liquid diet for 24 hours, restricted physical efforts, and other routine recommendations for this type of intervention.

The same postoperative medication protocol was adopted for both surgeries, which consisted of paracetamol (Tylenol® 750 mg, in Tablet) four times a day, for two days, in case of pain (15). No other drugs were allowed in postoperative phase. All patients were examined on fixed dates using standard methods and techniques. The measurements of edema, trismus, and pain were performed by a single-blind examiner (D.M.C.M.).

The edema was assessed by measuring facial width by five reference points from the mandibular angle: soft pogonion, buccal commissure, nose wing, eye outer corner, and tragus (17). The points were marked with a dermographic pen. A measuring tape was used to record the linear distances between the reference points. Three measurements were made for each distance, and a mean was extracted and recorded in millimeters, one hour before and 24 and 48 hours after surgery (15).

Trismus was assessed employing the upper and lower interincisal distance, measured with a pachymeter (18). The results were recorded in millimeters, one hour before and 24 and 48 hours after surgery (15).

Postoperative pain was assessed with a Visual Analogue Scale (VAS) and considering the number of analgesics consumed after each extraction. This information was recorded by the examiner 24 and 48 hours after surgery (15). In the measurement by VAS, patients measured pain by assigning a score from 0 to 10, where 0 corresponded to no pain and 10 corresponded to maximum intensity (18).

We adopted descriptors to predict drug permeability through the oral cavity based on the observation that ionized dexamethasone has significant oral permeability. The following descriptors were analyzed: Molecular Volume (MV), to assess molecular size; distribution coefficient (LogD), to assess solubility; the number of hydrogen donors (HBD); and the number of rotaTable bonds (nRotB), to determine the number of bonds which allow free rotation around themselves. All analyses were performed using the Molinspiration software package (http://www.molinspiration.com/).

- Statistical analyses

The primary exposure variable was dexamethasone (oral or intra-alveolar) administration route, and the covariates were represented by demographic data, tooth position classification, and surgical time.

Initially, descriptive statistics of categorical variables were performed, using frequency measures and numerical variables, using mean and standard deviation. The differences between the 24-hour and preoperative, 48-hour and preoperative, 48-hour, and 24-hour times were calculated for the numerical variables (face width and upper and lower interincisal distance). Distribution normality was assessed by the Shapiro-Wilk test. After this processing, the Student’s t-test was selected to compare the numerical variables (edema and trismus). The chi-square test analyzed the distribution of the categorical variable (postoperative pain) between the sides. We adopted a 5% (*p*<0.05) significance level. Data were analyzed using the SPSS statistical program (version 17.0).

## Results

Twenty-two individuals participated in the research, 10 females (45.5%) and 12 males (54.5%), aged 18-38 years (mean 29.0 ± 5.9). There were no differences between the operated sides regarding the mean surgical time for extraction (Table 1; *p*=0.662) and the position of the third molars according to the Winter and Pell & Gregory classification (Table 1; *p*> 0.05).

There were no significant differences between the administration routes in measuring facial edema between the preoperative and 24 and 48 postoperative hours considering the reference points of soft pogonion, oral commissure, nose wing, eye outer corner, and tragus (Table 2; *p*> 0.05).

Regarding trismus, there were also no significant differences between the operated sides concerning the maximum interincisal distance between the 24 hours and 48 hours and preoperative or between the two postoperative times (Table 2; *p*> 0.05).

Concerning the postoperative pain measured by the Visual Analogue Scale, no statistically significant differences were observed between the sides after 24 hours and 48 hours, nor in the number of analgesics consumed in the postoperative period at different times (Table 3; *p*> 0.05).

In silico prediction evaluation indicated that dexamethasone has favorable molecular characteristics for topical intra-alveolar administration. Table 4 shows the values ​​of the molecular descriptors of dexamethasone that indicate its permeability through oral tissues.

**Table 1:**
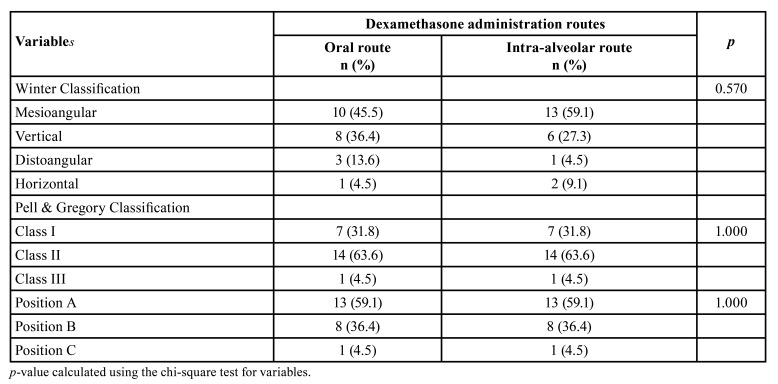
Distribution of variables related to the position of third molars between the study groups.

**Table 2:**
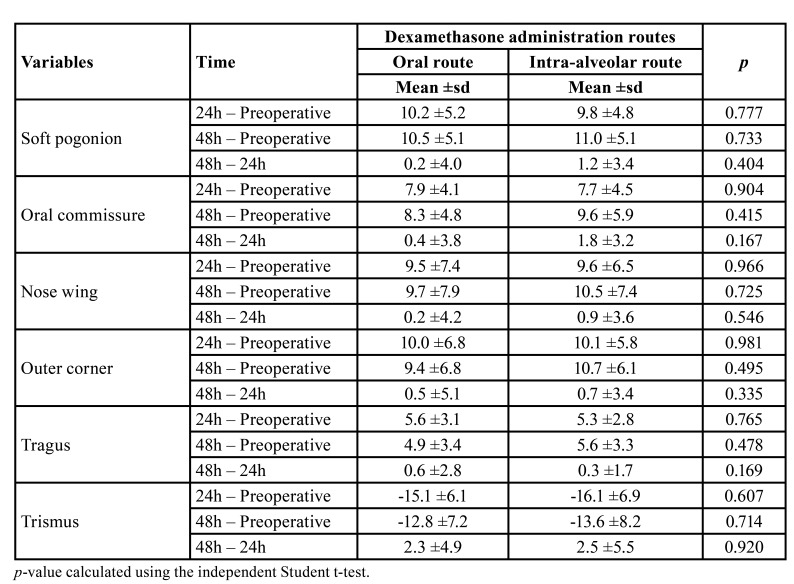
Mean and standard deviation of the variables referring to edema and trismus measurements at different times.

**Table 3:**
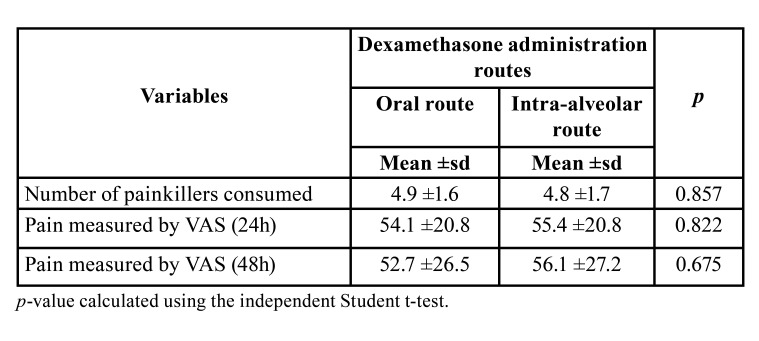
Distribution of postoperative pain variables.

**Table 4:**
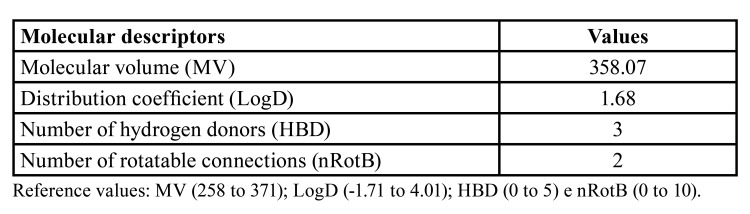
Values ​​of dexamethasone molecular descriptors to assess permeability through oral tissues.

## Discussion

This is the first randomized clinical trial to compare the administration of 4 mg dexamethasone by oral and intra-alveolar routes using the same drug formulation. The results showed no significant differences in the control of edema, trismus, and pain when comparing both approaches in impacted third molar surgeries. Also, *in silico* prediction of dexamethasone permeability through oral tissues showed that the intra-alveolar route is viable for the administration of dexamethasone. Altogether, the findings suggest that intra-alveolar administration of 4 mg dexamethasone powder may represent an alternative approach to prevent inflammation in third molar surgeries.

A randomized clinical trial was developed to compare the oral and intra-alveolar routes, as such study design is a gold standard for evaluating health treatments (19). Methodological approaches were used to control factors that could influence the inflammatory response, such as difficulty, time of surgery, and the patient’s characteristics. Furthermore, lower third molars impacted bilaterally, in equivalent positions, were included, according to the classification of Winter and Pell & Gregory, so that each patient was his control through the experimental split-mouth design. The difference in surgical time between each hemiarch was, at most, 5 minutes (12), and the study population consisted only of patients without any local or systemic alterations.

The effectiveness of topical intra-alveolar dexamethasone was previously suggested in a study comparing such administration route with the submucosal injection (12). The intra-alveolar use of dexamethasone was found to control edema, trismus, and post-surgical pain without increasing morbidity or causing side effects compared to submucosal drug use (12). In this study, the oral route was chosen as the control in comparisons with the intra-alveolar topical route of dexamethasone, since oral administration has been the most accepted, convenient (20), and most investigated route for the administration of dexamethasone in third molar surgeries (10).

Interestingly, the many properties of oral mucosa make it a viable site for administering drugs, such as accessibility, excellent blood supply, deviation of hepatic metabolism, and permeability profile (21). On the other hand, there are disadvantages such as the small area of the surface compared to the gastrointestinal mucosa and the continuous secretion of saliva, which can dilute the drug (11). In this study, dexamethasone powder was applied inside the alveolus immediately after extraction, and the suture was performed shortly after that. Thus, those disadvantages were not inconvenient for the use of the drug in this evaluation. However, considering that the oral mucosa epithelium acts as a barrier to protect the underlying tissues (22), we may speculate that the interior of the alveolus is an interesting place for the administration of the drug immediately after third molar surgery.

Besides the characteristics of the administration route, the permeability of the drug across biological membranes depends on structural and physicochemical properties, such as size, charge, lipophilicity, and hydrogen bonding capacity of the molecule (14). To our knowledge, this is the first study to analyze the permeability of dexamethasone through oral tissues by an *in silico* predictive model, which indicated that dexamethasone has promising characteristics for topical intra-alveolar administration. Some topical formulations of dexamethasone have been recommended to treat oral lesions, such as recurrent aphthous ulcerations (23), oral lichen planus (24), and oral vesicular lesions (25). In the studies by Majid and Mahmood (26) and Saravanan *et al*. (27), who used the topical submucosal administration of dexamethasone, no ulcerations or any other local side effect in the post-surgical evaluation were observed, suggesting the safe topical administration of dexamethasone in different formulations.

Regarding the dosage used in this study, several studies, including systematic reviews (10), have shown that oral 4 mg dexamethasone effectively controls inflammatory signs and symptoms in patients undergoing third molar extractions (2,28). Here, the same 4 mg dosage of dexamethasone was also used topically in this investigation, since the authors speculated that the onset of the drug at the site would be direct, without first-pass effects in the liver, therefore considering that it could be equally effective compared to its effects after oral administration.

Dexamethasone at 4 mg reduces edema in different administration regimens (2,26,28). In our study, we observed reduced edema without significant differences between the administration routes in the 24 and 48 hours postoperative times. Previous studies found similar results comparing the intra-alveolar (i.e., endo-alveolar) and submucosal routes of dexamethasone at 4 mg and 10 mg, respectively (12), and comparing 4 mg dexamethasone intravenously and 8 mg dexamethasone orally to control edema (3). The results suggest a similar efficacy for administering 4 mg dexamethasone by intra-alveolar route with other dosages and routes of administration.

The present study also assessed trismus after impacted third molar surgery. Boonsiriseth *et al*. (29), comparing oral and intramuscular administration of 8 mg dexamethasone, and Graziani *et al*. (12) comparing intra-alveolar/ endo-alveolar (4 mg or 10 mg) and submucosa (10 mg) administrations, found no significant differences in mouth opening limitation. Our results also showed no difference in reducing trismus between dexamethasone administered by the oral or intra-alveolar routes in the time points evaluated.

This investigation also found no significant differences of dexamethasone administration by oral and intra-alveolar routes of administration in the pain parameters, measured using the Visual Analogue Scale, ratified by the lack of a significant difference in the number of analgesics consumed in the post-surgical period for both administration routes. Previous studies have shown similar results, suggesting a similar analgesic effect on different dexamethasone administration routes (10,27). This effect could occur indirectly, due to reduced edema, through a significant decrease in the levels of prostaglandin E2 and thromboxane B2 in patients who received 4 mg dexamethasone with consequent pain reduction (30).

Considering that there were no significant differences in the clinical parameters of inflammation, the results suggest that professionals may choose one of the two routes of administration of 4 mg dexamethasone evaluated here in the extraction of impacted third molars.

In conclusion, 4 mg dexamethasone characteristics favor absorption through the oral mucosa, and its intra-alveolar administration had similar efficacy to oral administration in controlling postoperative inflammation symptoms of impacted lower third molars. The powder formulation used here effectively controlled the clinical inflammation symptoms in this research; however, future studies should develop a specific topical formulation for the intra-alveolar administration of dexamethasone.
